# Decentralized nursing education in Northern Norway: towards a sustainable recruitment and retention model in rural Arctic healthcare services

**DOI:** 10.3402/ijch.v72i0.22793

**Published:** 2013-11-25

**Authors:** Bente Norbye, Mari Wolff Skaalvik

**Affiliations:** Department of Health and Care Sciences, UiT The Arctic University of Norway, Tromsø, Norway

**Keywords:** bachelor in nursing, decentralized nursing education, off-campus education, recruitment, retention, rural areas

## Abstract

**Introduction:**

Decentralized nursing education (DNE) was established at Tromsø University College in 1990 and has since become a part of the bachelor programme in nursing at UiT The Arctic University of Norway. The objective of the study was to investigate whether and to what degree the first DNE programme established in Norway has contributed to recruitment and retention of registered nurses (RNs) in rural healthcare services.

**Methods:**

The quantitative survey took place in 2012. A questionnaire was distributed to 315 former students who had graduated from the DNE programme from 1994 to 2011.

**Results:**

The primary finding of this study is that the DNE successfully recruits students from rural areas of Northern Norway. Nearly, 87.5% have their first employment in community healthcare services. They continued to work in the rural areas and 85% still worked as nurses in 2012. The DNE programme has been successful regarding recruitment and retention of RNs to community healthcare services. Fifty-six percent have attended a variety of postgraduate programmes.

**Conclusion:**

The DNE programme demonstrates itself as a successful study model regarding recruitment and retention of RNs to rural and remote areas.

Internationally and nationally, there has been a focus on recruiting health professionals to rural and remote areas during 1980–1990 (1–4). Decentralized nursing education (DNE) is an important part of the educational system for recruiting qualified healthcare professionals to remote and rural areas as shown by Nilsen et al. (5) In Norway, Tromsø University College initiated the first DNE in 1990.

This study presents an overview of recruitment and retention of registered nurses (RNs) who graduated from a decentralized nursing programme in the county of Troms, Norway over a period of 20 years, including 11 admissions of students.

The Norwegian population is approximately 5 million and 90% of the population growth is in the larger towns and cities. In rural areas of Northern Norway, there is a population deficit and an ageing population in small rural municipalities (6). Recruitment and retention of healthcare professionals has been an issue in Northern Norway as well as being a global problem (2, 7–9).

Recruiting RNs to community healthcare services in Norway in general has also been an increasing problem. Nurses educated in the full-time 3-year nursing programme did not accommodate the need for nurses in the rural regions of Troms (10), as a majority of graduated RNs preferred to work in urban areas. Sustainable educational models to provide qualified health professionals in rural and remote areas (8) are still needed. *The Coordination Reform* (11), implemented in 2012, addresses expanded responsibilities and obligations in healthcare services at a municipal level. This requires qualified and sufficient staff as the municipalities are perceived as the key agency in providing healthcare services for the population.

A review by Dunbabin and Levitt (12) showed that students seeking admission to DNE programmes did so because they belonged to rural and remote areas and/or they had an interest in rural practice. It is stated that nursing students (NSs) recruited from rural and remote areas in the northern regions of Norway represent a notable difference in providing healthcare services in such areas because they are familiar with the local culture and specific healthcare needs in the population (5). Medical students from Northern Norway with a significant affiliation to the region improved the recruitment of doctors in their region of Northern Norway (2).

## Norwegian healthcare system

The Norwegian healthcare system is organized through specialist healthcare services and community healthcare services. Each of the 19 counties in Norway offer specialist healthcare in highly specialized hospitals. Four hundred and thirty Norwegian municipalities offer community healthcare services, with the main services as care for the elderly in nursing homes, home-based nursing and preventive healthcare. District Medical Centres are established as part of inter-municipal services. Community healthcare services is the main catchment area for RNs from the DNE, hence indicating the importance of relevant postgraduate education for RNs living and working in rural and remote areas. As in the rest of the Western world, care for the elderly is not a preferred occupation and this has impeded recruitment to this part of the healthcare system (13) and lead to a high turnover.

## Norwegian nursing education

The Norwegian nursing education is a 3-year bachelor (BA) programme covering 180 European Credit Transfer System (ECTS) points and is approved in European Community countries. Clinical practice and theoretical studies each equal 90 ECTS. In Norway 28 University Colleges and Universities, including both public and private university colleges, deliver the programme. The number of places for NSs is approximately 13,800 (13).

## The DNE programme

**Fig. 1:**
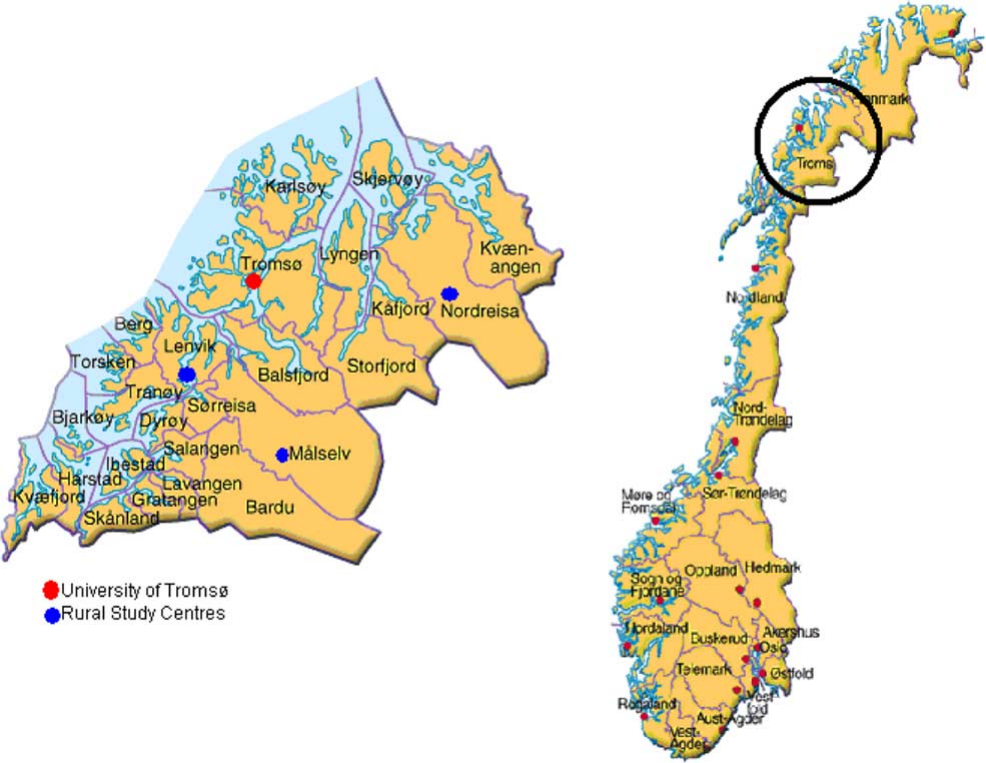
Norway, extracted the county of Troms with its study centres.

Nursing education, decentralized from urban health science centres into rural areas, was established for the first time in the United States in 1974 (1). In 1990, as a national pilot, Tromsø University College piloted a decentralized nursing programme to provide RNs to the rural and remote regions in a county in Northern Norway (14). The objective was twofold. One aim was to offer higher education to candidates living in rural areas with responsibilities and obligations in their home community that restricted them from moving away from their home community for educational purposes. The second aim was to strengthen the nursing workforce in selected municipalities. The DNE was organized as a part-time, 4-year programme, delivered to students from the rural areas in the county of Troms (Fig. 1). Nursing lecturers from the geographical areas were recruited (14, 15) with the main responsibility of teaching and supervising the NSs in this model's off-campus learning activities, in cooperation with and with support from on-campus staff giving lectures. The programme was organized according to the national guidelines for nursing education (16) but with expanded study time and including internet-based communication technology such as videoconferencing, a learning management system to support the students’ self-studies and off-campus learning activities. This blended learning approach (17), with a mix of traditional learning activities and on-line activities, was chosen to provide a flexible and dynamic study model making it possible for the students to combine personal responsibilities with their studies. The curriculum was offered primarily off-campus through work in established study groups supervised by university lecturers. Some courses were introduced in week sessions conducted on-campus, although never more than twice per semester. Supervised clinical placements were offered locally except for medical and surgical clinical placements in the second study year. For these placements, the students had to stay in a nearby town with a hospital.

Students admitted to the DNE had to document their connection to a rural municipality. This was part of the initial partnership between 11 selected municipalities, later extended to 18 municipalities, and the University of Tromsø. The students had to meet the general qualification requirements for studying at a university or university college. The rural municipalities initiated the partnership between the university and the municipalities due to the vacancies of qualified RNs. In 1989, these municipalities had 57 vacancies out of 188 available nursing positions (10). Based on the students’ connection to a local, rural municipality one assumed that they would work as nurses in their municipalities after graduation.

Initially, the DNE project was met with scepticism from the Norwegian nurses’ organization (NNO) and university colleges who were concerned that the DNE could be perceived as a second-class nursing education. This has been accounted for in the curriculum as it is outlined in accordance with the national guidelines for nursing education in Norway (16). Among the sceptics, it was suggested that the need for nurses in remote and rural municipalities would quickly be saturated, leaving graduated nurses in those areas unemployed.

The first admission of students to the DNE in 1990 was based on a report (10) that concluded that there was an obvious need to recruit nurses to rural areas in Northern Norway. Former strategies to recruit nurses to these areas, mainly including financial incentives, had proven unsuccessful as educated nurses left the rural areas after a few years. The situation can be described in terms of a lack of RNs and a lack of continuity in the community healthcare services including home-based care, nursing homes and primary healthcare.

As the first class graduated as nurses from the decentralized education in 1994, 356 RNs in 11 admissions have graduated by 2011.

After 20 years of delivering the DNE, it is important to obtain systematic knowledge about recruitment and the retention of RNs to the rural areas comprised by the DNE. Community healthcare services in Norway are facing new challenges consisting of an ageing population and an increased number of persons with chronic diseases, combined with advances in medical expertise and healthcare technologies resulting in more people surviving life-threatening conditions (1, 8, 11). Hence, the need for healthcare services and qualified healthcare professionals within community healthcare services will increase. The Norwegian government has implemented *The Coordination Reform* (11) to address the changing demands in healthcare services. Healthcare services at a community-based level are perceived as the key agency for the future organization of services distributed across traditional boundaries, both at the municipal level and in the specialist healthcare service (11).

This article presents the results of a questionnaire survey on recruitment and retention of RNs who graduated from the DNE in Northern Norway from 1990–2011.

The objective of this study was to investigate whether and to what degree the DNE programme from 1990 to 2011 has contributed to the recruitment and retention of RNs in rural healthcare services.

## Methods

This is a quantitative survey with descriptive statistics.

### Sample and participants

The participants invited to contribute to this study attended the DNE at UiT The Arctic University of Norway, from 1990 to 2011. The former students were asked to respond to a questionnaire posted to their private addresses. From the sample of 356 former students, 41 questionnaires were returned by the postal service as the addressees had moved without leaving a new address, hence 315 questionnaires were posted.

Altogether, 233 former students returned the questionnaire. Due to missing participants, the findings cannot be considered representative for the entire population of graduated RNs according to the DNE within the selected time span.

### The questionnaire

The questionnaire was developed by the 2 researchers, as no validated questionnaires were found on the study topic. The questionnaire consists of background variables and 5 main subject areas with sub-questions addressing geographical locations, employment, work areas, postgraduate education and career paths. Responses were limited to the options in the questionnaire. Before finalizing the questionnaire, a panel of university lecturers assessed it. A pilot study was conducted among 11 healthcare students at a university to pre-test the questionnaire before implementation of the study. The pre-test resulted in minor refinements. The survey was conducted in December 2011.

### Data collection

The data were collected from NSs graduated from the DNE at a UiT The Arctic University of Norway. A research assistant administered posting and coding the questionnaires to ensure that the researchers had no knowledge of the names of the participants who completed the questionnaires. The questionnaire was posted to the participants with an information letter and a pre-paid return envelope. One reminder was posted to non-responders 2 weeks after the originally given reply date.

### Statistical analysis

Data were analyzed using IBM SPSS statistics 20.0 (www-01.ibm.com/software/analytics/spss/) with cross-sectional and descriptive univariate statistics for multiple variables; frequency, mean, percent, range with cross-sectional and descriptive data (18).

### Ethics approval

The study was approved by the Norwegian Social Science Data Services (NSD), approval number 27459. Head of Department at the university's Department of Health and Care Sciences allowed the study to take place by releasing the names and addresses of the former decentralized NSs. Initially, the participants were informed about the purpose of the study in an explanatory letter and they were asked to participate by filling in and returning the questionnaire. Their informed consent was given by completing and returning the questionnaire in the attached envelope. Confidentiality and anonymity were guaranteed.

## Results

### Response rate

Of 315 questionnaire-receiving graduated RNs from the DNE from 1990 to 2011, 233 answered the questionnaire, resulting in a response rate of 73.9%.

### Recruitment of NSs to the DNE

The admission to the DNE from 1990 to 2011 has doubled from 1990 35 to 2011 70 in each class. The average age of the respondents at the start of the nursing education was 31.2 years ranging from 19 to 47 years, with little changes over this period. In 1990, the average age was 29.1 years and in 2007, it had increased slightly to 30.8. Among the respondents (n = 233), there were 218 female (93.6%) and 15 male (6.4%) graduated nurses. This is in accordance with the gender differences in Norwegian nursing education where approximately 10% of the NSs are male (19).

The NSs were predominantly recruited from 18 rural municipalities (n=180) in the county of Troms, and from 1 urban town (n=35). The rural municipalities have a population ranging from under 1,000 up to 12,000; the urban town has 70,000 citizens. It was possible to indicate multiple reasons for applying to the DNE. The main reasons cited included the possibility of combining family responsibilities and work commitments. Other reasons for attending the DNE were given as: interest in the nursing profession, work possibilities after graduation, and the possibility of attending higher education while living in a rural area (Table I).

**Table I:** Reasons for applying a DNE

Reason	Frequency	Percentage
Family responsibility	142	60.9
Work commitment	34	14.6
Study close to home	84	36.1
Possibility to study part-time	131	56.2
Others	21	

### First employment after graduation

After graduation, the predominant employment for 87.5% of the graduated RNs was within community healthcare services and 22.3% in specialist healthcare services. Other employments (5.6%) included ambulance service, air ambulance service, private healthcare and occupational healthcare services.

The proportion of participants’ employment after graduation shows a decline, but not more than what can be expected in a 20-year period (Table II). Eighty-five percent (n = 199) of the respondents worked as nurses in 2012. Out of 24 not working as nurses, 11 still worked in the healthcare services— 4 in the ambulance service, 2 as midwives and 5 as leaders of services. Seven have retired from employment and 6 have left the nursing profession. Reasons for leaving the nursing profession were given as health issues and other employment opportunities.

**Table II:** Current employment for nurses completing the DNE in 1994–2011

	Frequency		
	
	Current employment	Not working as nurses	Total
1994	18	5	23
1995	13	5	18
1996	15	6	21
1997	23	2	25
2001	20	4	24
2002	22	3	25
2005	23	3	26
2006	16	0	16
2007	19	2	21
2009	20	1	21
2011	11	1	12
Total	200	33	233

**Fig. 2:**
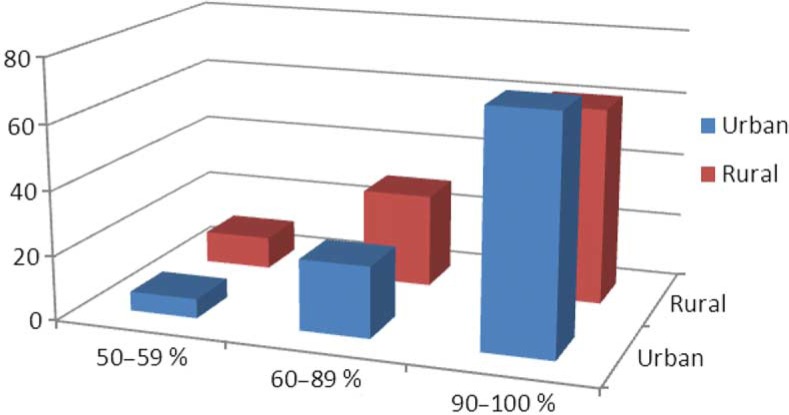
Initial employment after completing DNE.

The majority of the respondents (73%) started their career as RNs in permanent positions, 21% started as substitutes and 6% started in temporary positions. There was no significant difference between respondents from urban and rural areas as far as choice of occupation area (Fig. 2).

### Retention in healthcare services

The survey shows that nurses educated in the DNE have initial work in municipal healthcare services and a majority continued to work in the rural areas. The retention rate among nurses was 81.6% after graduation. These nurses have become a stable workforce in the community healthcare services. In the rural areas of the county in question, there are also 3 outpatient District Medical Centres offering specialist healthcare services in psychiatric and somatic care (Table III).

**Table III:** Employment and workstations in 2012 (n = 200)

	Frequency	Percentage
Community healthcare services	136	68.0
Nursing home	59	29.5
Home-based care	44	22.0
Psychiatric home-based care	9	4.5
Primary health nurse	7	3.5
Doctors’ office	8	4.0
Emergency service	9	4.5
Specialist healthcare services	53	26.5
Hospital ward's somatic care, including outpatient clinics	30	15.0
Psychiatric outpatient clinic	23	11.5
Other	11	5.5

### Postgraduate education

In Norway, inclusive of Northern Norway, postgraduate education is offered for all specialities within specialist healthcare, as well as for preventive healthcare, psychiatry and gerontology. Among the respondents (n=233), 56% (n=131) of the graduated RNs have attended 146 postgraduate programmes organized as both part- and full-time studies.

Postgraduate education conducted by the respondents is mainly directed towards clinical specialization. According to community healthcare services, 52% (n=97) of the respondents have completed postgraduate education in geriatric nursing, dementia and psychiatric healthcare. Another 16% (n=29) attended programmes originally designed for employment in specialist healthcare such as intensive care, paediatric care, anaesthetics, cancer care and operating theatre nursing. A lower percentage (0.9%) have attended administrative and leadership programmes, although 20% (n=41) of the respondents worked as leaders at the time of the survey. Some of the respondents (n=16) had completed 2 or more postgraduate programmes. The majority of the respondents who had completed postgraduate education had done this as part-time and/or were decentralized students (80%).

Studies on a master level in nursing were conducted by 3 respondents. Master studies had not been offered according to a decentralized model at the time of the survey.

## Discussion

The results of this survey show that RNs who graduated from the DNE represent a stable workforce in rural Arctic municipalities. This finding is in accordance with Nilsen et al. (5), and it is interpreted as a result of a structure for admission to the DNE that valued applicants with rural bonds and connections. The fact that the average age was 30 or more demonstrates that the DNE reached adult candidates searching for education and professional competence, making it possible for them to continue living and working as professionals in a remote and rural municipality. Recruiting nurses to rural areas is not only a Northern Norwegian issue, as approximately 62% of the world's population live in rural areas, served by only 38% of the total nursing workforce (20). The candidates from this DNE programme express that the possibility of combining family responsibilities and work commitments provided them the possibility of attending nursing education. Those who wish to live in remote and rural areas experience challenges in obtaining higher education due to geographical distance to higher education institutions as well as practical and financial matters.

## A decentralized model in a rural setting

The DNE was organized as a part-time study at local study centres in order to accommodate the combination of family and working responsibilities. This is in agreement with conclusions reached in a study by Kavern and Webb (21) stating that curriculum redesigns are needed to accommodate the integration of family life and studies for mature women. The study by Nilsen et al. (5) illustrates the success of educating inhabitants in their local environment. That 97.3% of the respondents in this study completed the study within the prescribed time frame proves that by extending the ordinary full-time programme by 1 year, it was feasible for the majority of the respondents to combine both personal and study requirements. This was accommodated by patterning the learning process with a combination of overview lectures on-campus, supervised group sessions locally and organized self-studies on an individual as well as interpersonal basis (22). In conclusion, the students’ study progress was supported by a flexible study programme.

## Future implications for community health service

It is stated that education for specialist healthcare has been predominant at the expense of primary healthcare services, both on a national and international level (11, 23). National government recommendations (11) include a requirement for patients to receive proper treatment at the right place and at the right time. A main feature of the implementation of *The Coordination Reform* is the provision of healthcare services at the municipal level with a competent workforce in its service.

The main catchment area for students educated in the DNE is municipal healthcare services. Findings in this study show a high retention rate, as the RNs still work in their home municipality up to 20 years after graduation and first employment. Thus, DNE provides community healthcare with a stable workforce. Evidently, it shows a lower turnover of nurses compared with international findings (24, 25). Community healthcare service is the cornerstone in healthcare. By recruiting employees with a local affiliation wishing to live and work in these rural and Arctic surroundings, continuity and a sustainable healthcare service is made possible.

Statistical projections in Norway (6) estimate that there will be a shortage of 28,200 nurses in 2035 and the required increase in healthcare services will be in community healthcare services. According to *The Coordination Reform* (11), it is vital for the healthcare services to strengthen municipal healthcare, and based on the findings in this study the DNE is seen as contributing to this. However, the need for RNs with specialized education such as palliative care and specialized knowledge in chronic diseases will increase in remote and rural areas (11). Therefore, education programmes facilitating this competence in remote and rural areas are necessary. Among the respondents, 56% of the respondents have attended postgraduate education and 117 respondents have conducted postgraduate programmes organized as part-time students in DNEs. This is interpreted as a positive attitude towards educational programmes, corresponding with the way their BA training was organized.

The fact that the majority of the respondents who participated in this study entered working life in full-time positions refuted the fear of saturation of the labour market for RNs. This is due to the politically based recognition of community healthcare services (11). Recruiting healthcare professionals to rural and remote areas is a worldwide challenge. The results from this study demonstrate that a DNE contributes to meeting this challenge and can be of interest for other rural areas, as well as countries with similar challenges.

It is still a challenge for universities to provide education as off-campus programmes both on a BA and on a Masters level. Part-time programmes are often delivered to attract more students but not especially designed for students with family commitments and work obligations. In Norway, most part-time programmes are delivered on-campus, with time and cost demands for the students. Universities have an obligation to provide professional education that safeguards society's needs. Education models adapted to diverse groups of students must therefore be a priority. This will secure the recruitment of NSs to rural and remote areas in accordance with goals stated in *The Coordination Reform* (11) as well as international requirements (23).

### Study limitations

The findings in this study are limited due to the lack of data about non-receivers of the questionnaire as well as non-respondents.

From the findings in this study, further investigations regarding study models supporting recruitment and retention of RNs to rural and remote areas are recommended for policy development on both a national and international level.

## Conclusion

From the results in this study, more than 20 years of offering nursing education through a DNE has proven to contribute to a sustainable healthcare service in a Northern Norwegian context. Scepticism regarding competence and possible unemployment has been proven wrong. Current and future changes in community healthcare services, with a transfer of responsibility in treatment and care from specialist care to municipal healthcare, puts pressure on remote and rural municipalities regarding recruiting and retaining qualified healthcare professionals. Development of future curriculum models must be the subject for systematic evaluation in order to ensure that qualified RNs living in rural areas are afforded possibilities for postgraduate education, enabling them to meet future needs in the municipal healthcare services. Decentralized healthcare education is one way of contributing to this as it contributes to professional competence vital for the implementation of current healthcare policies in Norway. Finally, the DNE is a success as it primarily provides women in rural areas with the possibility to fulfil competing demands and to more fully develop their potential to contribute to their communities.
